# Binge drinking differentially affects cortical and subcortical microstructure

**DOI:** 10.1111/adb.12493

**Published:** 2017-01-20

**Authors:** Laurel S. Morris, Nicholas G. Dowell, Mara Cercignani, Neil A. Harrison, Valerie Voon

**Affiliations:** ^1^ Behavioural and Clinical Neuroscience Institute University of Cambridge Cambridge UK; ^2^ Department of Psychology University of Cambridge Cambridge UK; ^3^ Department of Psychiatry Brighton and Sussex Medical School Brighton UK; ^4^ Department of Psychiatry University of Cambridge, Addenbrooke's Hospital Cambridge UK

## Abstract

Young adult binge drinkers represent a model for endophenotypic risk factors for alcohol misuse and early exposure to repeated binge cycles. Chronic or harmful alcohol use leads to neurochemical, structural and morphological neuroplastic changes, particularly in regions associated with reward processing and motivation. We investigated neural microstructure in 28 binge drinkers compared with 38 matched healthy controls. We used a recently developed diffusion magnetic resonance imaging acquisition and analysis, which uses three‐compartment modelling (of intracellular, extracellular and cerebrospinal fluid) to determine brain tissue microstructure features including neurite density and orientation dispersion index (ODI). Binge drinkers had reduced ODI, a proxy of neurite complexity, in frontal cortical grey matter and increased ODI in parietal grey matter. Neurite density was higher in cortical white matter in adjacent regions of lower ODI in binge drinkers. Furthermore, binge drinkers had higher ventral striatal grey matter ODI that was positively correlated with binge score. Healthy volunteers showed no such relationships. We demonstrate disturbed dendritic complexity of higher‐order prefrontal and parietal regions, along with higher dendritic complexity of a subcortical region known to mediate reward‐related motivation. The findings illustrate novel microstructural abnormalities that may reflect an infnce of alcohol bingeing on critical neurodevelopmental processes in an at‐risk young adult group.

## Introduction

Binge drinking, the rapid intake of alcohol in short bursts of time, is a serious public health issue in the United Kingdom and United States, costing an estimated £4.9bn/year to British society (Francesconi 2015). This common pattern of alcohol intake has the highest prevalence in young adults (Kuntsche, Rehm & Gmel 2004; Grucza, Norberg & Bierut 2009), during a time of heightened risk‐taking, impulsivity, neural (ventral striatal) response to rewards (Hill *et al.* 2000; Braams *et al.* 2015), and crucially, alongside ongoing neurodevelopment. Young adults who binge drink but not those who drink without this pattern (Miller *et al.* 2007) partake in other detrimental behaviours, ultimately linking binge drinking with accidents, violence, suicide and alcohol‐induced liver disease (Mathurin & Deltenre 2009; Stolle, Sack & Thomasius 2009; Nutt & Rehm 2014) as well as heightened substance abuse or dependence (Chassin, Pitts & Prost 2002).

While acute alcohol consumption can locally modulate neurotransmission, more chronic use has counter‐adaptive effects on neural systems (Vengeliene *et al.* 2008), which is further perturbed by states of withdrawal (Rolland *et al.* 2011) (Rossetti & Carboni 1995). Local neuromodulatory changes associated with harmful alcohol use also promote both small and large‐scale structural and functional changes in the longer term. Adolescent and young adult binge drinkers feature a range of grey and white matter volume changes that coincide with cognitive impairments. In teenagers who developed regular alcohol use patterns in the previous year, reductions in cortical grey matter volumes have been reported, in particular of the dorsolateral prefrontal cortex (dlpfc) and premotor cortex (Luciana *et al.* 2013). Reduced cerebellar grey matter volume has also been associated with severity of binge drinking in a large sample of healthy teenagers (Lisdahl *et al.* 2013). Contrastingly, higher left dlpfc grey matter volume has also been reported in this group, associated with past alcohol consumption (Doallo *et al.* 2014). We have also recently reported larger ventral striatal volumes in binge drinkers (Howell *et al.* 2013). Binge drinkers also display functional changes in these regions; activity in the dorsal prefrontal cortex during a spatial working memory task is reduced (Squeglia *et al.* 2011), and resting‐state functional connectivity of the ventral striatum is reduced and associated with impulsivity (Morris *et al.* 2015a, 2015b).

While informative, assessments of grey matter volume provide little indication of morphological or microstructural differences that may be present that are associated with neuroplastic restructuring in response to patterns of alcohol use. We therefore employed a recently developed diffusion MRI technique that provides higher specificity of microstructural characteristics than conventional diffusion tensor imaging to examine the relationship between binge drinking and microstructure. Diffusion of water, which is normally isotropic, is restricted in the brain by tissue boundaries such as axonal membranes and as such, different microstructural environments can be assessed. Previous models of white matter use a diffusion ellipsoid or diffusion tensor to capture the features of white matter fibre bundles that restrict movement of water. The ball‐and‐stick model (Behrens *et al.* 2003) represents the intracellular water diffusion component as cylinders with zero radius (stick) and extracellular diffusion as isotropic and unrestricted (ball). These models assume one single orientation of fibres, which limits analysis to coherently organized fibre bundles like the corpus callosum while grey matter displays substantial dispersion of fibre orientations. More model‐based methods using geometric models of microstructure to predict the MR signal produced by water diffusion can explicitly represent the dispersion of axon orientation, expected in grey matter. We use neurite orientation dispersion density imaging (NODDI) with microstructural modelling that has a more direct relationship with axonal orientation distribution (Jespersen *et al.* 2012), neurite density and dendritic architecture (Jespersen *et al.* 2010).

This approach is based on the ‘hindered and restricted model’ of white matter water diffusion and uses three‐compartment modelling (Panagiotaki *et al.* 2012) for three distinct tissue microstructural environments that each uniquely effect water diffusion. The differentiation of such water forms provides a basis for depiction of microstructural features using diffusion MRI. Firstly, the intracellular fraction ultimately provides a measure of how dispersed fibres are, indicating the complexity of neurite or dendritic branching expected in grey matter [orientation dispersion index (ODI)]. Secondly, the extracellular fraction is equivalent to myelinated fibre bundles expected in white matter (neurite density). Thirdly, the cerebrospinal fluid (CSF) space is where diffusion of water is isotropic (Zhang *et al.* 2012). As such, measures of neurite density and complexity can be obtained, which provide a more fine‐grained microstructural approach and have good scan–rescan reproducibility (Tariq *et al.* 2012). With traditional DTI measures, like fractional anisotropy, it remains unclear whether lower fractional anisotropy equates to lower coherence of white matter fibres. However, with the current analysis, the ODI captures sprawling dendritic processes, detailing grey matter complexity (Zhang *et al.* 2012). ODI is consistent with Golgi staining of dendritic processes (Jespersen *et al.* 2012) and microscopic grey matter dendritic architecture (Jespersen *et al.* 2010). Indeed, these microstructural features have previously been associated with the hierarchy of computations performed by increasingly higher‐level cortical structures (Jacobs *et al.* 2001), goal‐directed behaviour (Morris *et al.* 2015a, 2015b), lower age (Nazeri *et al.* 2015) and resting‐state functional network connectivity (Nazeri *et al.* 2015).

We characterized changes in microstructural features across the brain of young adult binge drinkers and examined alcohol use severity and the specific pattern of binge drinking. As neural microstructural maturation trajectories persist into the early 20s (Lebel *et al.* 2008), we examined a young adult population. In line with our previous findings of larger ventral striatal grey matter volume in binge drinkers (Howell *et al.* 2013), we expected that binge drinkers would have higher orientation dispersion in this region, potentially marking neural proliferation associated with excessive alcohol use or motivation for reward.

## Materials and Methods

### Participants

Young adult binge drinkers and healthy volunteers were recruited from community‐based advertisements in East Anglia. Binge drinkers inclusion criteria was based on the National Institute on Alcoholism and Alcohol Abuse [(NIAAA) 2004] diagnostics: >8/>6 alcohol units consumption (men/women) within a 2 hour period at least once a week. Subjects had to have been ‘drunk’ at least once per week for the previous 6 months and reported an intention to get drunk. Subjects were carefully questioned on their patterns of alcohol consumption and last alcohol binge consumption prior to testing. There was no upper limit for amount of binges or times ‘being drunk’ over the previous 6 months, but alcohol dependence was exclusionary. Healthy volunteers were made up of drinkers (non‐binge) and non‐drinkers. Psychiatric disorders including substance addictions were screened with the Mini International Neuropsychiatric Interview (Sheehan *et al.* 1998). Subjects were excluded if they had a major psychiatric disorder, substance addiction (including alcohol and excluding nicotine) or medical illness or were on psychotropic medications. Subjects were included if they were 18 years of age or over, were right‐handed only and had no history of regular or current use of other substances.

All participants completed the National Adult Reading Test (Nelson 1982) to assess verbal IQ, the Beck Depression Inventory (Beck *et al.* 1961) for depressive symptoms and the UPPS‐P scale for impulsivity (Whiteside & Lynam 2001). The Alcohol Use Disorders Test (AUDIT) (Saunders *et al.* 1993) was used to assess general alcohol use severity. The Alcohol Use Questionnaire was used to assess the pattern of alcohol use, the last binge, frequency of alcohol use, amount of alcohol intake and speed of drinking (Townshend & Duka 2002). From this, a ‘binge score’ can be calculated, which is less susceptible to self‐report estimation distortions (Townshend & Duka 2002). This score incorporates the speed of drinking, the amount of times being ‘drunk’ in the previous 6 months and the percentage of times that an individual drinks to get drunk. This provides a measure of the *pattern* of drinking as opposed to simply the amount of alcohol consumed. Therefore, one may have relatively low alcohol intake but a high binge score. Participants provided written informed consent and were compensated for their time. The study was approved by the University of Cambridge Research Ethics Committee. Grey matter volumetric data have previously been reported in a subset of this sample, showing a sexually dimorphic pattern of volume change in binge drinkers (Kvamme *et al.* 2016).

### Neurite orientation dispersion and density imaging

The current analysis of diffusion MRI data is based on the hindered and restricted model of white matter water diffusion using three‐compartment modelling (Panagiotaki *et al.* 2012) for three distinct tissue microstructural environments. Firstly, the intracellular fraction shows restricted diffusion with a non‐Gaussian pattern of water displacement, in which diffusion is bounded by restricted geometries like axonal membranes. The total signal is thus a composite of diffusion restriction by a cylinder with a given orientation and weighted by all cylinders oriented in that direction. While previous models use a single, parallel orientation parameter, here, we used a Watson distribution (spherical analogue of a Gaussian distribution) to signify axons dispersing about a central orientation, which can range from highly parallel to highly dispersed. This ultimately provides a measure of ODI, or how dispersed fibres are, indicating the complexity of neurite or dendritic branching. Secondly, the extracellular fraction shows hindered diffusion and a Gaussian anisotropic displacement, in which water diffusion is hindered by glial and cell body (soma) membranes. Thirdly, the CSF space is where water diffusion is unhindered and isotropic (Zhang *et al.* 2012). The differentiation of such water forms provides a basis for depiction of microstructural features using diffusion MRI. ODI captures sprawling dendritic processes, detailing grey matter complexity (Zhang *et al.* 2012) that is consistent with Golgi staining of dendritic processes (Jespersen *et al.* 2012) and microscopic grey matter dendritic architecture (Jespersen *et al.* 2010).

We acquired NODDI data from 38 healthy volunteers and 28 binge drinkers. Data were acquired with a Siemens 3T Tim Trio scanner using a 32‐channel head coil at the Wolfson Brain Imaging Centre at the University of Cambridge with the following parameters: TE = 128 milliseconds; TR = 11 300 milliseconds; planar FOV = 192 mm × 192 mm; 96 matrix with 2‐mm voxel and 2‐mm slice thickness. There were 63 slices (b = 0 volumes and diffusion weighted data in two shells, b‐values: 2850 and 700 seconds/mm^2^ with 65 and 33 directions, respectively). A NODDI microstructural model was computed and fitted to the data using the NODDI toolbox for Matlab (Zhang *et al.* 2012) (http://www.nitrc.org/projects/noddi_toolbox). Resulting parameter maps were normalized to MNI space with ANTS software (http://stnava.github.io/ANTs/). ODI parameter maps were masked to standard grey matter and neurite density to standard white matter templates. Parameter maps for both measures were entered into independent samples *t*‐test analysis to compare between groups, controlling for age and gender. Whole‐brain corrected family‐wise error (FWE) *p* < 0.05 was considered significant for these group comparisons and thresholded at cluster extent of <10 contiguous voxels to remove contributions from small spurious clusters. For correlations between microstructure and drinking severity, these whole brain parameter maps were entered into correlation analysis with AUDIT and binge scores, separately for healthy volunteers and binge drinkers and whole‐brain FWE *p* < 0.05 was considered significant.

To examine whether there were group differences in the ventral striatum specifically based on a priori hypotheses, we computed small volume corrected (SVC) region of interest (ROI) analysis (*p* < 0.05) for ventral striatum. The ventral striatal anatomical ROI, as used previously in other studies (Murray *et al.* 2008), was hand drawn using MRIcro based on the definition of the ventral striatum by Martinez *et al.* (2003).

## Results

### Participant characteristics

We acquired data from 38 healthy volunteers (24 women; current/occasional smokers = 1/3) and 28 binge drinkers (11 women; current/occasional smokers, 5/3; days since last binge, 2.85, 1.89 SD). See Table 1 for demographic data. There were no signification group differences in age (*p* = 0.697), gender (*p* = 0.102), verbal IQ (*p* = 0.321) or years of education (*p* = 0.438).

**Table 1:** Demographic data.

		Mean	SD	*p*
Gender (F/M)	HV	24/14		0.102
	BD	11/17		
Age	HV	23.69	3.85	0.697
	BD	22.03	4.47	
Verbal IQ	HV	115.54	7.16	0.321
	BD	117.21	4.58	
BDI	HV	6.973	7.407	0.208
	BD	9.636	8.37	
AUDIT	HV	4.333	3.119	<0.001
	BD	16.75	4.529	
Binge score	HV	7.545	6.035	<0.001
	BD	33.376	15.371	
Percent drunk	HV	0.112	0.224	<0.001
	BD	0.47	0.291	
Amount drunk	HV	1.5	3.121	<0.001
	BD	15.889	14.175	
UPPS total	HV	127.5	16.246	0.004
	BD	141.091	16.622	
Sensation seeking	HV	32.5	6.701	0.025
	BD	36.409	5.17	
Positive urgency	HV	27.594	4.785	0.036
	BD	31.682	7.828	

Data are presented for 38 healthy volunteers and 28 binge drinkers.

AUDIT = Alcohol Use Disorders Test; BD = binge drinkers; BDI = Beck Depression Inventory; F/M = female/male; HV = healthy volunteers; *p* = independent samples *t*‐test *p* value; UPPS = urgency, premeditation, perseverance, sensation seeking, and positive urgency impulsive behavior scale; SD = standard deviation.

Binge drinkers had significantly higher ‘binge score’, AUDIT score and had significantly more days that they were ‘drunk’ and percentage that they would drink to get drunk in the last 12 months (Table 1). Of the healthy volunteers, 17 reported zero times ‘being drunk’ in the past 6 months. Binge drinkers also had higher total UPPS total scores, higher sensation seeking and higher positive urgency (Table 1). There was a significant difference between smoking status (*p* = 0.033) between the two groups. The two groups did not significantly differ in depressive symptoms (*p* = 0.208).

### Neurite orientation dispersion and density

**Figure 1:**
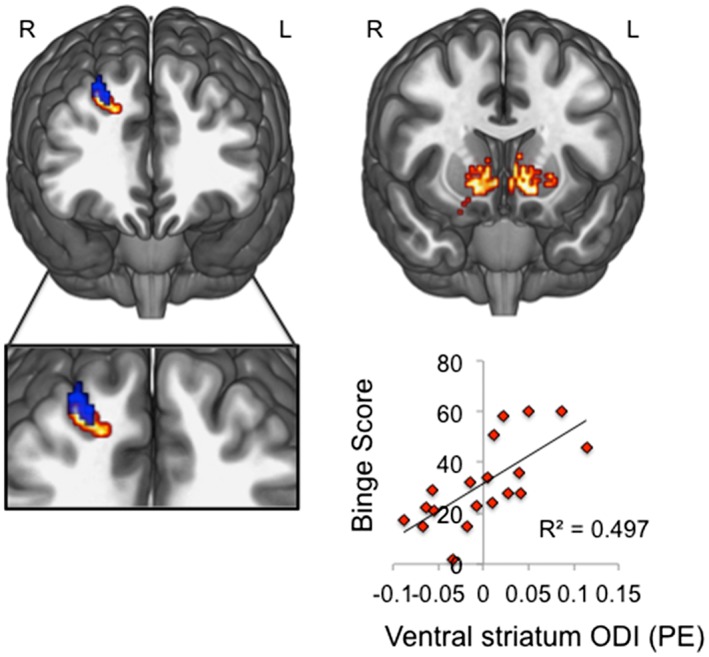
Lower dorsolateral prefrontal cortex but higher ventral striatal orientation dispersion in binge drinkers. Left: clusters illustrate regions of significantly higher white matter neurite density (red) and lower grey matter orientation dispersion index (ODI, blue) in dlpfc of binge drinkers compared with healthy volunteers (displayed at *p* < 0.001 uncorrected threshold for visualization purposes). Right: small volume corrected family‐wise error *p* < 0.05 analyses revealed that binge drinkers had reduced ODI in ventral striatum compared with matched controls. Left sided peak ventral striatal ODI (peak coordinates, xyz = −6 12 0) positively correlated with binge score in binge drinkers. Images are displayed on a standard MNI152 template; R, right; L, left

Microstructural maps derived from the NODDI acquisition and analysis for example healthy volunteer are depicted in the Supporting Information Fig. 1. Binge drinkers had lower grey matter ODI in several regions, including right dlpfc and higher adjacent white matter neurite density (whole‐brain FWE corrected *p* < 0.05, see Table 2 for further statistics) (Fig. 1). Binge drinkers also had decreased ODI and increased neurite density in regions throughout the parietal cortex (Table 2) (Supporting Information Fig. 2). There were no differences between men and women across both groups in ODI or neurite density (FWE *p* > 0.05). We specifically examined group differences in ventral striatal microstructure given our previous findings of volumetric changes in ventral striatal in binge drinkers (Howell *et al.* 2013) using ROI analysis. Binge drinkers had higher bilateral ventral striatal orientation dispersion compared with healthy volunteers (peak coordinate at left ventral striatum, xyz = −6 12 0; ventral striatal SVC FWE *p* = 0.003; *Z* = 4.62) (Fig. 1).

**Table 2:** Statistics of group differences for neurite density and orientation dispersion index.

	p(FWE‐corr)	K	Z	x	y	z
Orientation Dispersion Index						
HV > BD						
Right inferior parietal cortex	<0.001	14	>8	50	−64	22
Right superior frontal gyrus (DLPFC)	<0.001	27	7.75	24	40	38
Left middle occipital gyrus	<0.001	12	7.06	−28	−82	36
			6.44	−32	−78	30
	<0.001	10	6.94	−28	−74	44
Right postcentral gyrus	<0.001	22	7.05	34	−34	58
Left superior parietal lobule	<0.001	10	6.42	−24	−58	62
BD > HV						
Right angular gyrus	<0.001	26	7.84	50	−60	26
	<0.001	15	7.33	−40	−76	32
	<0.001	12	6.43	42	−58	40
	<0.001	10	5.74	−42	−62	20
Left superior parietal lobule	<0.001	22	7.19	−22	−64	52
Right supramarginal gyrus	<0.001	22	6.95	56	−42	40
Left inferior parietal lobule	<0.001	12	6.89	−40	−40	46
	<0.001	16	6.06	30	−42	56
Neurite density						
HV > BD						
Right angular gyrus	<0.001	19	7.77	46	−58	26
	<0.001	12	6.61	54	−48	30
Left middle occipital gyrus	<0.001	16	6.59	−38	−74	32
Right postcentral gyrus	<0.001	15	6.08	32	−38	48
BD > HV						
Inferior parietal cortex	<0.001	20	>8	48	−64	22
	<0.001	13	6.82	34	−84	22
			5.68	36	−76	22
	<0.001	10	6.04	44	−76	12
Right superior frontal gyrus (DLPFC)	<0.001	27	7.71	24	38	38
Left middle frontal gyrus (DLPFC)	<0.001	21	7.44	−28	40	26
	<0.001	20	6.6	−22	30	38
Right supramarginal gyrus	<0.001	29	7.57	54	−44	32
			6.06	54	−46	42
Right middle frontal gyrus	<0.001	19	6.33	38	12	48
Right superior frontal gyrus	<0.001	12	6.18	26	−8	58

BD = binge drinkers; DLPFC = dorsolateral prefrontal cortex; HV = healthy volunteers; K = cluster size; p(FWE‐corr) = whole brain (*p* < 0.05) family‐wise error corrected *p* value; xyz = peak voxel coordinates; Z = *z*‐score.

As there were widespread ODI and neurite density differences between groups, both higher (parietal cortex) and lower ODI (dlpfc) in binge drinkers, it is important to determine whether relationships between microstructure and behaviour can be established across the whole brain, which will illuminate the specificity and relevance of the microstructural differences. Thus, we examined the relationship between microstructural features and drinking severity using AUDIT and the ‘binge score’ obtained from the Alcohol Use Questionnaire (Townshend & Duka 2002). There were no significant correlations between whole brain ODI and drinking severity (binge score or AUDIT) at whole‐brain FWE corrected *p* < 0.05). However, when restricting the analysis to ventral striatum, we find that binge drinkers show a positive correlation between ventral striatal ODI and binge score (bilateral ventral striatum SVC FWE *p* = 0.011; *Z* = 4.32, peak xyz = 12 14–8) (Fig. 1) but not AUDIT (*p* > 0.05). No significant correlations between ventral striatal ODI and drinking severity were observed in healthy volunteers. To explore the specificity of these findings, we controlled for AUDIT in the binge score correlation, which did not affect the significance of the findings (positive correlation between right ventral striatum ODI and binge score controlling for AUDIT in binge drinkers: SVC FWE *p* = 0.012).

As measures of impulsivity were significantly different between groups behaviourally (UPPS total, positive urgency and sensation seeking), we conducted correlations between microstructural parameter maps and measures of impulsivity in an exploratory manner across groups. There were no significant correlations between whole brain ODI or neurite density and impulsivity (all whole‐brain FWE corrected *p* < 0.05). As smoking status differed between the two groups, smoking was added as a covariate of no interest to the analyses. Smoking did not affect the whole‐brain group difference findings (Supporting Information Table 1) or the SVC findings for ventral striatum (bilateral ventral striatum SVC FWE *p* = 0.004; *Z* = 4.54, peak xyz = −14 10 0).

## Discussion

We used a recently developed diffusion MRI acquisition and analysis to determine brain tissue microstructure features in young adult binge drinkers. This is the first study demonstrating reduced ODI, a proxy of neurite complexity, in the frontal cortical grey matter of binge drinkers and increased ODI in parietal cortex. Furthermore, neurite density was higher in cortical white matter in adjacent regions of lower ODI in binge drinkers. While there is a weak relationship between ODI and neurite density (Zhang *et al.* 2012), the current study suggests their joint disturbance in binge drinkers. Cortical microstructural changes were not specifically related to alcohol use severity or binge drinking patterns in the cortex; however, the ventral striatum showed higher ODI that was associated with binge severity in binge drinkers. The link between ventral striatal dendritic complexity and binge drinking score but not general alcohol use severity suggests that the behavioural binge pattern of alcohol intake (rapid and frequent bouts of heavy drinking) rather than general alcohol use severity is associated with ventral striatal neurite integrity.

The current measure of dendritic complexity has previously been associated with age (Nazeri *et al.* 2015) and the hierarchy of neural computations (Jacobs *et al.* 2001). Hetero‐modal cortical regions required for downstream processing of information show more complex dendrite and spine features than primary and uni‐modal cortical regions (Jacobs *et al.* 2001). This complexity is captured by the current method and is consistent with both Golgi staining of dendritic processes (Jespersen *et al.* 2012) and microscopic detailing of grey matter dendritic architecture (Jespersen *et al.* 2010). Thus, lower dlpfc ODI in binge drinkers may reflect a reduction in the highly dispersed dendritic tree organization expected in high‐level cortical regions. Indeed, this converges with observations that binge drinking in young adults is associated with significant attentional and executive functioning deficits subserved by dlpfc (Scaife & Duka 2009; Parada *et al.* 2012; Mota *et al.* 2013). Furthermore, alcohol craving is associated with dlpfc activity (Olbrich *et al.* 2006), and modulation of dlpfc with transcranial direct current stimulation reduces craving in individuals with alcohol use disorders (Boggio *et al.* 2008). The finding of higher ODI in the parietal cortex of binge drinkers was unexpected. The parietal lobe plays a diverse role in cognition, directing attentional control (Shulman *et al.* 1999; Corbetta *et al.* 2000), task switching (Sohn *et al.* 2000) and response inhibition (de Zubicaray *et al.* 2000). While previous studies have implicated dlpfc (Luciana *et al.* 2013) and ventral striatal (Howell *et al.* 2013) volume changes in binge drinkers, changes in parietal cortex have also been suggested in alcohol use disorders. Alcohol‐dependent individuals show reduced parietal grey matter volumes associated with lifetime alcohol use (Fein *et al.* 2002; Rando *et al.* 2011) and spatial processing (Fein *et al.* 2009), and alcohol‐dependent women show reduced parietal activity during a spatial working memory task (Tapert *et al.* 2001). Together, while the direction of microstructural change requires further exploration and determination, the current findings suggest a disturbance in fronto‐parietal integrity in binge drinkers. It will be important for future studies to define the functional relevance of these microstructural changes by specifically examining attentional and executive processing alongside detailed microstructural analyses.

Previous studies of ventral striatal volume in individuals with AUD have engendered mixed results. There have been reports of lower ventral striatal volume (Sullivan *et al.* 2005; Makris *et al.* 2008; Wrase *et al.* 2008); however, a recent report demonstrated an association between higher incidence of familial AUD and greater left ventral striatal volume (Cservenka *et al.* 2015). We have also recently shown that binge drinkers have enlarged ventral striatal volume (Howell *et al.* 2013) influenced by gender (Kvamme *et al.* 2016). These findings relating to grey matter volume are mixed; however, the current measure is distinct from volume.

Distinct from measures of volume, the current finding of higher ventral striatal ODI may reflect a neuroplastic capacity. For example, chronic psychostimulant use increases dendritic arborization in the ventral striatum of rodents (Robinson & Kolb 2004), and plasticity of nucleus accumbens mediates the reinforcing effects of ethanol (Rassnick, Pulvirenti & Koob 1992). Furthermore, while we do not currently examine grey matter volume, we have previously demonstrated in a subset of the same binge drinkers a sexually dimorphic pattern of volume change wherein binge drinking men show higher and binge drinking women show lower ventral striatum volume relative to their healthy counterparts (Kvamme *et al.* 2016). In contrast to the current study, the same paper reported no group differences in volumetric data, suggesting a dissociation between these measures. While further longitudinal studies are necessary to elucidate the neuroplastic ventral striatal changes associated with patterns of alcohol use, these findings suggest that cortical microstructural may be broadly associated with harmful alcohol use.

We did not find a relationship between impulsivity and whole brain microstructure or ventral striatal microstructure. Impulsivity has been suggested as an endophenotypic marker for subsequent compulsive substance use or dependence (Everitt & Robbins 2005; Belin *et al.* 2008; Robbins *et al.* 2012). As such, relating ventral striatal microstructure to impulsivity might suggest it as a potential endophenotypic marker. However, at least for ventral striatal ODI, the differences may be a result of alcohol use rather than the underlying behavioural factor of impulsivity.

We highlight disturbances in neurite microstructure in binge drinkers in regions implicated in higher‐order attentional and executive functioning and reward‐related motivation. Whether the current neural findings are a cause or effect of alcohol exposure requires further longitudinal studies. Interestingly, adolescents who initiate alcohol use show a reduction in grey matter in the right middle frontal gyrus, overlapping with dlpfc, compared with those who did not initiate alcohol use (Luciana *et al.* 2013), suggesting a potential pre‐morbid role of dlpfc structural integrity. Alternatively, alcohol or drug use may interfere with critical neurodevelopmental stages that persist into young adulthood. We caution that underlying neurobiological differences cannot be directly inferred by the current technique but that there is some broad indication of microstructural change. ODI has been previously associated with higher‐level processing (Jacobs *et al.* 2001) and because we report ODI changes in cortical regions involved with executive behavioural control; we highlight the potential pathological and functional relevance of this measure. Further studies directly examining the relationships between cortical ODI and flexible behavioural control are certainly warranted.

### Financial and Disclosure

This study was funded by the Wellcome Trust Fellowship grant for VV (093705/Z/10/Z). V.V. and N.A.H. are Wellcome Trust (WT) intermediate Clinical Fellows. L.S.M. is in receipt of an MRC studentship. The BCNI is supported by a WT and MRC grant. The remaining authors declare no competing financial interests.

### Authors Contribution

All authors contributed to the study design and acquisition of the data. LSM, NGD analysed the data. LSM drafted the manuscript. All authors assisted the interpretation of the findings, critically reviewed the content and approved the final version for publication.

## Supporting information


**Supplementary Table 1.** Statistics of group differences for neurite density and orientation dispersion index, controlled for smoking status. Abbreviations: p(FWE‐corr), whole brain (*P* < 0.05) family‐wise error corrected P value; K, cluster size; Z, Z‐score; xyz, peak voxel coordinates; BD, binge drinkers; HV, healthy volunteers; DLPFC, dorsolateral prefrontal cortex.
**Supplementary Figure 1.** Example microstructure parameters. Microstructure parameters for a single healthy subject are presented. Abbreviations: ODI, orientation dispersion index; ICVF, intracellular volume fraction, for neurite density; CSF, cerebrospinal fluid.
**Supplementary Figure 2.** Regions of reduced orientation dispersion index in binge drinkers compared to healthy volunteers. Binge drinkers had reduced grey matter orientation dispersion index (ODI) in right dorsolateral prefrontal cortex (dlpfc) and in distinct regions throughout the parietal cortex (whole‐brain family‐wise error (FWE) corrected *p* < 0.05, see Table 1 for further statistics).

Supporting Info Item

## References

[adb12493-bib-0001] (NIAAA), N. I. o. A. A. a. A ( 2004). NIAAA council approves definition of binge drinking. NIAAA Newsletter 3.

[adb12493-bib-0002] Beck AT , Erbaugh J , Ward CH , Mock J , Mendelsohn M (1961) An inventory for measuring depression. Arch Gen Psychiatry 4:561–571. 13688369 10.1001/archpsyc.1961.01710120031004

[adb12493-bib-0003] Behrens TE , Woolrich MW , Jenkinson M , Johansen‐Berg H , Nunes RG , Clare S , Matthews PM , Brady JM , Smith SM (2003) Characterization and propagation of uncertainty in diffusion‐weighted MR imaging. Magn Reson Med 50:1077–1088. 14587019 10.1002/mrm.10609

[adb12493-bib-0004] Belin D , Mar AC , Dalley JW , Robbins TW , Everitt BJ (2008) High impulsivity predicts the switch to compulsive cocaine‐taking. Science 320:1352–1355. 18535246 10.1126/science.1158136 PMC2478705

[adb12493-bib-0005] Boggio PS , Sultani N , Fecteau S , Merabet L , Mecca T , Pascual‐Leone A , Basaglia A , Fregni F (2008) Prefrontal cortex modulation using transcranial DC stimulation reduces alcohol craving: a double‐blind, sham‐controlled study. Drug Alcohol Depend 92:55–60. 17640830 10.1016/j.drugalcdep.2007.06.011

[adb12493-bib-0006] Braams BR , van Duijvenvoorde AC , Peper JS , Crone EA (2015) Longitudinal changes in adolescent risk‐taking: a comprehensive study of neural responses to rewards, pubertal development, and risk‐taking behavior. J Neurosci 35:7226–7238. 25948271 10.1523/JNEUROSCI.4764-14.2015 PMC6605271

[adb12493-bib-0007] Chassin L , Pitts SC , Prost J (2002) Binge drinking trajectories from adolescence to emerging adulthood in a high‐risk sample: predictors and substance abuse outcomes. J Consult Clin Psychol 70:67–78. 11860058

[adb12493-bib-0008] Corbetta M , Kincade JM , Ollinger JM , McAvoy MP , Shulman GL (2000) Voluntary orienting is dissociated from target detection in human posterior parietal cortex. Nat Neurosci 3:292–297. 10700263 10.1038/73009

[adb12493-bib-0009] Cservenka A , Gillespie AJ , Michael PG , Nagel BJ (2015) Family history density of alcoholism relates to left nucleus accumbens volume in adolescent girls. J Stud Alcohol Drugs 76:47–56. 25486393 PMC4263780

[adb12493-bib-0010] de Zubicaray GI , Andrew C , Zelaya FO , Williams SCR , Dumanoir C (2000) Motor response suppression and the prepotent tendency to respond: a parametric fMRI study. Neuropsychologia 38:1280–1291. 10865104 10.1016/s0028-3932(00)00033-6

[adb12493-bib-0011] Doallo S , Cadaveira F , Corral M , Mota N , Lopez‐Caneda E , Holguin SR (2014) Larger mid‐dorsolateral prefrontal gray matter volume in young binge drinkers revealed by voxel‐based morphometry. PLoS One 9. 10.1371/journal.pone.0096380 PMC4008532 24789323

[adb12493-bib-0012] Everitt BJ , Robbins TW (2005) Neural systems of reinforcement for drug addiction: from actions to habits to compulsion. Nat Neurosci 8:1481–1489. 16251991 10.1038/nn1579

[adb12493-bib-0013] Fein G , Di Sclafani V , Cardenas VA , Goldmann H , Tolou‐Shams M , Meyerhoff DJ (2002) Cortical gray matter loss in treatment‐naive alcohol dependent individuals. Alcohol Clin Exp Res 26:558–564. 11981133 PMC2435064

[adb12493-bib-0014] Fein G , Shimotsu R , Chu R , Barakos J (2009) Parietal gray matter volume loss is related to spatial processing deficits in long‐term abstinent alcoholic men. Alcohol Clin Exp Res 33:1806–1814. 19645730 10.1111/j.1530-0277.2009.01019.x PMC2755629

[adb12493-bib-0015] Francesconi M (2015) The cost of binge drinking. CEPR Discussion Paper No. DP10412.

[adb12493-bib-0016] Grucza RA , Norberg KE , Bierut LJ (2009) Binge drinking among youths and young adults in the United States: 1979–2006. J Am Acad Child Adolesc Psychiatry 48:692–702. 19465879 10.1097/CHI.0b013e3181a2b32f PMC2862553

[adb12493-bib-0017] Hill SY , Shen S , Lowers L , Locke J (2000) Factors predicting the onset of adolescent drinking in families at high risk for developing alcoholism. Biol Psychiatry 48:265–275. 10960157 10.1016/s0006-3223(00)00841-6

[adb12493-bib-0018] Howell NA , Worbe Y , Lange I , Tait R , Irvine M , Banca P , Harrison NA , Bullmore ET , Hutchison WD , Voon V (2013) Increased ventral striatal volume in college‐aged binge drinkers. PLoS One 8:e74164. 24086317 10.1371/journal.pone.0074164 PMC3785474

[adb12493-bib-0019] Jacobs B , Schall M , Prather M , Kapler E , Driscoll L , Baca S , Jacobs J , Ford K , Wainwright M , Treml M (2001) Regional dendritic and spine variation in human cerebral cortex: a quantitative Golgi study. Cereb Cortex 11:558–571. 11375917 10.1093/cercor/11.6.558

[adb12493-bib-0020] Jespersen SN , Bjarkam CR , Nyengaard JR , Chakravarty MM , Hansen B , Vosegaard T , Ostergaard L , Yablonskiy D , Nielsen NC , Vestergaard‐Poulsen P (2010) Neurite density from magnetic resonance diffusion measurements at ultrahigh field: comparison with light microscopy and electron microscopy. Neuroimage 49:205–216. 19732836 10.1016/j.neuroimage.2009.08.053 PMC2862296

[adb12493-bib-0021] Jespersen SN , Leigland LA , Cornea A , Kroenke CD (2012) Determination of axonal and dendritic orientation distributions within the developing cerebral cortex by diffusion tensor imaging. IEEE Trans Med Imaging 31:16–32. 21768045 10.1109/TMI.2011.2162099 PMC3271123

[adb12493-bib-0022] Kuntsche E , Rehm J , Gmel G (2004) Characteristics of binge drinkers in Europe. Soc Sci Med 59:113–127. 15087148 10.1016/j.socscimed.2003.10.009

[adb12493-bib-0023] Lebel C , Walker L , Leemans A , Phillips L , Beaulieu C (2008) Microstructural maturation of the human brain from childhood to adulthood. Neuroimage 40:1044–1055. 18295509 10.1016/j.neuroimage.2007.12.053

[adb12493-bib-0024] Lisdahl KM , Thayer R , Squeglia LM , McQueeny TM , Tapert SF (2013) Recent binge drinking predicts smaller cerebellar volumes in adolescents. Psychiatry Res‐Neuroim 211:17–23. 10.1016/j.pscychresns.2012.07.009 PMC3670762 23154095

[adb12493-bib-0025] Luciana M , Collins PF , Muetzel RL , Lim KO (2013) Effects of alcohol use initiation on brain structure in typically developing adolescents. Am J Drug Alcohol Abuse 39:345–355. 24200204 10.3109/00952990.2013.837057 PMC4076828

[adb12493-bib-0026] Makris N , Oscar‐Berman M , Jaffin SK , Hodge SM , Kennedy DN , Caviness VS , Marinkovic K , Breiter HC , Gasic GP , Harris GJ (2008) Decreased volume of the brain reward system in alcoholism. Biol Psychiatry 64:192–202. 18374900 10.1016/j.biopsych.2008.01.018 PMC2572710

[adb12493-bib-0027] Martinez D , Slifstein M , Broft A , Mawlawi O , Hwang DR , Huang Y , Cooper T , Kegeles L , Zarahn E , Abi‐Dargham A , Haber SN , Laruelle M (2003) Imaging human mesolimbic dopamine transmission with positron emission tomography. Part II: amphetamine‐induced dopamine release in the functional subdivisions of the striatum. J Cereb Blood Flow Metab 23:285–300. 12621304 10.1097/01.WCB.0000048520.34839.1A

[adb12493-bib-0028] Mathurin P , Deltenre P (2009) Effect of binge drinking on the liver: an alarming public health issue? Gut 58:613–617. 19174416 10.1136/gut.2007.145573

[adb12493-bib-0029] Miller JW , Naimi TS , Brewer RD , Jones SE (2007) Binge drinking and associated health risk behaviors among high school students. Pediatrics 119:76–85. 17200273 10.1542/peds.2006-1517

[adb12493-bib-0030] Morris LS , Kundu P , Baek K , Irvine MA , Mechelmans DJ , Wood J , Harrison NA , Robbins TW , Bullmore ET , Voon V (2015a) Jumping the gun: mapping neural correlates of waiting impulsivity and relevance across alcohol misuse. Biol Psychiatry 79:499–507. 26185010 10.1016/j.biopsych.2015.06.009 PMC4764648

[adb12493-bib-0031] Morris LS , Kundu P , Dowell N , Mechelmans DJ , Favre P , Irvine MA , Robbins TW , Daw N , Bullmore ET , Harrison NA , Voon V (2015b) Fronto‐striatal organization: defining functional and microstructural substrates of behavioural flexibility. Cortex 74:118–133. 26673945 10.1016/j.cortex.2015.11.004 PMC4729321

[adb12493-bib-0032] Mota N , Parada M , Crego A , Doallo S , Caamano‐Isorna F , Holguin SR , Cadaveira F , Corral M (2013) Binge drinking trajectory and neuropsychological functioning among university students: a longitudinal study. Drug Alcohol Depend 133:108–114. 23791027 10.1016/j.drugalcdep.2013.05.024

[adb12493-bib-0033] Murray GK , Corlett PR , Clark L , Pessiglione M , Blackwell AD , Honey G , Jones PB , Bullmore ET , Robbins TW , Fletcher PC (2008) Substantia nigra/ventral tegmental reward prediction error disruption in psychosis. Mol Psychiatry 13:239–276. 17684497 10.1038/sj.mp.4002058 PMC2564111

[adb12493-bib-0034] Nazeri A , Chakravarty MM , Rotenberg DJ , Rajji TK , Rathi Y , Michailovich OV , Voineskos AN (2015) Functional consequences of neurite orientation dispersion and density in humans across the adult lifespan. J Neurosci 35:1753–1762. 25632148 10.1523/JNEUROSCI.3979-14.2015 PMC4308611

[adb12493-bib-0035] Nelson H (1982) National Adult Reading Test. Windsor, UK: NFER‐Nelson.

[adb12493-bib-0036] Nutt DJ , Rehm J (2014) Doing it by numbers: a simple approach to reducing the harms of alcohol. J Psychopharmacol 28:3–7. 24399337 10.1177/0269881113512038

[adb12493-bib-0037] Olbrich HM , Valerius G , Paris C , Hagenbuch F , Ebert D , Juengling FD (2006) Brain activation during craving for alcohol measured by positron emission tomography. Aust N Z J Psychiatry 40:171–178. 16476136 10.1080/j.1440-1614.2006.01765.x

[adb12493-bib-0038] Panagiotaki E , Schneider T , Siow B , Hall MG , Lythgoe MF , Alexander DC (2012) Compartment models of the diffusion MR signal in brain white matter: a taxonomy and comparison. Neuroimage 59:2241–2254. 22001791 10.1016/j.neuroimage.2011.09.081

[adb12493-bib-0039] Parada M , Corral M , Mota N , Crego A , Rodriguez Holguin S , Cadaveira F (2012) Executive functioning and alcohol binge drinking in university students. Addict Behav 37:167–172. 21996093 10.1016/j.addbeh.2011.09.015

[adb12493-bib-0040] Rando K , Hong KI , Bhagwagar Z , Li CS , Bergquist K , Guarnaccia J , Sinha R (2011) Association of frontal and posterior cortical gray matter volume with time to alcohol relapse: a prospective study. Am J Psychiatry 168:183–192. 21078704 10.1176/appi.ajp.2010.10020233 PMC3668974

[adb12493-bib-0041] Rassnick S , Pulvirenti L , Koob GF (1992) Oral ethanol self‐administration in rats is reduced by the administration of dopamine and glutamate receptor antagonists into the nucleus‐accumbens. Psychopharmacology (Berl) 109:92–98. 1365677 10.1007/BF02245485

[adb12493-bib-0042] Robbins TW , Gillan CM , Smith DG , de Wit S , Ersche KD (2012) Neurocognitive endophenotypes of impulsivity and compulsivity: towards dimensional psychiatry. Trends Cogn Sci 16:81–91. 22155014 10.1016/j.tics.2011.11.009

[adb12493-bib-0043] Robinson TE , Kolb B (2004) Structural plasticity associated with exposure to drugs of abuse. Neuropharmacology 47:33–46. 15464124 10.1016/j.neuropharm.2004.06.025

[adb12493-bib-0044] Rolland B , Karila L , Guardia D , Cottencin O (2011) Pharmaceutical approaches of binge drinking. Curr Pharm Des 17:1333–1342. 21524262 10.2174/138161211796150792

[adb12493-bib-0045] Rossetti ZL , Carboni S (1995) Ethanol withdrawal is associated with increased extracellular glutamate in the rat striatum. Eur J Pharmacol 283:177–183. 7498307 10.1016/0014-2999(95)00344-k

[adb12493-bib-0046] Saunders JB , Aasland OG , Babor TF , de la Fuente JR , Grant M (1993) Development of the alcohol use disorders identification test (AUDIT): WHO collaborative project on early detection of persons with harmful alcohol consumption–II. Addiction 88:791–804. 8329970 10.1111/j.1360-0443.1993.tb02093.x

[adb12493-bib-0047] Scaife JC , Duka T (2009) Behavioural measures of frontal lobe function in a population of young social drinkers with binge drinking pattern. Pharmacol Biochem Behav 93:354–362. 19497334 10.1016/j.pbb.2009.05.015

[adb12493-bib-0048] Sheehan DV , Lecrubier Y , Sheehan KH , Amorim P , Janavs J , Weiller E , Hergueta T , Baker R , Dunbar GC (1998) The Mini‐International Neuropsychiatric Interview (M.I.N.I.): the development and validation of a structured diagnostic psychiatric interview for DSM‐IV and ICD‐10. J Clin Psychiatry 59:22–33. ;quiz 34‐57. 9881538

[adb12493-bib-0049] Shulman GL , Ollinger JM , Akbudak E , Conturo TE , Snyder AZ , Petersen SE , Corbetta M (1999) Areas involved in encoding and applying directional expectations to moving objects. J Neurosci 19:9480–9496. 10531451 10.1523/JNEUROSCI.19-21-09480.1999 PMC6782891

[adb12493-bib-0050] Sohn MH , Ursu S , Anderson JR , Stenger VA , Carter CS (2000) The role of prefrontal cortex and posterior parietal carter in task switching. Proc Natl Acad Sci U S A 97:13448–13453. 11069306 10.1073/pnas.240460497 PMC27244

[adb12493-bib-0051] Squeglia LM , Schweinsburg AD , Pulido C , Tapert SF (2011) Adolescent binge drinking linked to abnormal spatial working memory brain activation: differential gender effects. Alcohol Clin Exp Res 35:1831–1841. 21762178 10.1111/j.1530-0277.2011.01527.x PMC3183294

[adb12493-bib-0052] Stolle M , Sack PM , Thomasius R (2009) Binge drinking in childhood and adolescence: epidemiology, consequences, and interventions. Dtsch Arztebl Int 106:323–328. 19547732 10.3238/arztebl.2009.0323 PMC2689602

[adb12493-bib-0053] Sullivan EV , Deshmukh A , De Rosa E , Rosenbloom MJ , Pfefferbaum A (2005) Striatal and forebrain nuclei volumes: contribution to motor function and working memory deficits in alcoholism. Biol Psychiatry 57:768–776. 15820234 10.1016/j.biopsych.2004.12.012

[adb12493-bib-0054] Tapert SF , Brown GG , Kindermann SS , Cheung EH , Frank LR , Brown SA (2001) fMRI measurement of brain dysfunction in alcohol‐dependent young women. Alcohol Clin Exp Res 25:236–245. 11236838

[adb12493-bib-0055] Tariq M , Schneider T , Alexander DC , Wheeler‐Kingshott C , Zhang H (2012) Scan‐rescan reproducibility of neurite microstructure estimates using NODDI. Medical Image Understanding and Analysis 2012: Proceedings of the 16th Conference on Medical Image Understanding and Analysis: 255–261.

[adb12493-bib-0056] Kvamme TL , Schmidt C , Strelchuk D , Chang‐Webb YC , Baek K , Voon V (2016) Sexually dimorphic brain volume interaction in college‐aged binge drinkers. Neuroimage Clin 10:310–317. 26900571 10.1016/j.nicl.2015.12.004 PMC4724035

[adb12493-bib-0057] Townshend JM , Duka T (2002) Patterns of alcohol drinking in a population of young social drinkers: a comparison of questionnaire and diary measures. Alcohol Alcohol 37:187–192. 11912076 10.1093/alcalc/37.2.187

[adb12493-bib-0058] Vengeliene V , Bilbao A , Molander A , Spanagel R (2008) Neuropharmacology of alcohol addiction. Br J Pharmacol 154:299–315. 18311194 10.1038/bjp.2008.30 PMC2442440

[adb12493-bib-0059] Whiteside SP , Lynam DR (2001) The five factor model and impulsivity: using a structural model of personality to understand impulsivity. Personal Individ Differ 30:669–689.

[adb12493-bib-0060] Wrase J , Makris N , Braus DF , Mann K , Smolka MN , Kennedy DN , Caviness VS , Hodge SM , Tang L , Albaugh M , Ziegler DA , Davis OC , Kissling C , Schumann G , Breiter HC , Heinz A (2008) Amygdala volume associated with alcohol abuse relapse and craving. Am J Psychiatry 165:1179–1184. 18593776 10.1176/appi.ajp.2008.07121877

[adb12493-bib-0061] Zhang H , Schneider T , Wheeler‐Kingshott CA , Alexander DC (2012) NODDI: practical in vivo neurite orientation dispersion and density imaging of the human brain. Neuroimage 61:1000–1016. 22484410 10.1016/j.neuroimage.2012.03.072

