# Supplementary material for: Binge drinking differentially affects cortical and subcortical microstructure
Source: Addict Biol. 2017 Jan 20;23(1):403–11. doi: 10.1111/adb.12493 (PMC5811821; doi:10.1111/adb.12493)
Supplement: Supplementary file 1 — Supporting Info Item [file ADB-23-403-s001.zip › supp.docx]

Supplementary Materials

Supplementary Table 1. Statistics of group differences for neurite density and orientation dispersion index, controlled for smoking status.

|  | p(FWE-corr) | K | Z | x | y | z |
| --- | --- | --- | --- | --- | --- | --- |
| ***Orientation Dispersion Index*** | |  |  |  |  |  |
| ***HV>BD*** |  |  |  |  |  |  |
| Right Inferior Parietal Cortex | <0.001 | 13 | >8 | 50 | -64 | 22 |
| Right Superior Frontal Gyrus (DLPFC) | <0.001 | 24 | 7.47 | 24 | 40 | 38 |
| Left Middle Occipital Gyrus | <0.001 | 11 | 7.11 | -28 | -82 | 36 |
|  |  |  | 6.31 | -32 | -78 | 30 |
|  | <0.001 | 8 | 6.96 | -28 | -74 | 44 |
| Right Postcentral Gyrus | <0.001 | 18 | 6.8 | 34 | -34 | 58 |
| Left Superior Parietal Lobule | <0.001 | 9 | 6.37 | -24 | -58 | 62 |
| ***BD>HV*** |  |  |  |  |  |  |
| Right Angular Gyrus | <0.001 | 25 | 7.69 | 50 | -60 | 26 |
|  | <0.001 | 12 | 7.04 | -40 | -76 | 32 |
|  | <0.001 | 11 | 6.25 | 42 | -58 | 40 |
| Left Superior Parietal Lobule | <0.001 | 20 | 6.93 | -22 | -64 | 52 |
| Right SupraMarginal Gyrus | <0.001 | 21 | 6.85 | 56 | -42 | 40 |
| Left Inferior Parietal Lobule | <0.001 | 10 | 6.74 | -40 | -40 | 46 |
|  | <0.001 | 16 | 6.18 | 30 | -42 | 56 |
| ***Neurite Density*** | |  |  |  |  |  |
| ***HV>BD*** |  |  |  |  |  |  |
| Right Angular Gyrus | <0.001 | 17 | 7.55 | 46 | -58 | 26 |
|  | <0.001 | 12 | 6.45 | 54 | -48 | 30 |
| Left Middle Occipital Gyrus | <0.001 | 16 | 6.3 | -38 | -74 | 32 |
| ***BD>HV*** |  |  |  |  |  |  |
| Inferior Parietal Cortex | <0.001 | 19 | >8 | 48 | -64 | 22 |
|  | <0.001 | 13 | 6.96 | 34 | -84 | 22 |
| Right Superior Frontal Gyrus (DLPFC) | <0.001 | 24 | 7.47 | 24 | 38 | 38 |
| Left Middle Frontal Gyrus (DLPFC) | <0.001 | 19 | 7.2 | -28 | 40 | 26 |
|  | <0.001 | 14 | 6.38 | -22 | 30 | 38 |
| Right SupraMarginal Gyrus | <0.001 | 25 | 7.35 | 54 | -44 | 32 |
|  |  |  | 6.19 | 54 | -46 | 42 |
| Right Middle Frontal Gyrus | <0.001 | 16 | 6.14 | 38 | 12 | 48 |
| Right Superior Frontal Gyrus | 0.001 | 3 | 6.01 | 26 | -8 | 58 |

Supplementary Table 1. Statistics of group differences for neurite density and orientation dispersion index, controlled for smoking status. Abbreviations: p(FWE-corr), whole brain (P<0.05) family-wise error corrected P value; K, cluster size; Z, Z-score; xyz, peak voxel coordinates; BD, binge-drinkers; HV, healthy volunteers; DLPFC, dorsolateral prefrontal cortex.

Supplementary Figure Legends

Supplementary Figure Legends

Supplementary Figure 1. Example microstructure parameters. Microstructure parameters for a single healthy subject are presented. Abbreviations: ODI, orientation dispersion index; ICVF, intracellular volume fraction, for neurite density; CSF, cerebrospinal fluid.

Supplementary Figure 2. Regions of reduced orientation dispersion index in binge drinkers compared to healthy volunteers. Binge-drinkers had reduced grey matter orientation dispersion index (ODI) in right dorsolateral prefrontal cortex (dlpfc) and in distinct regions throughout the parietal cortex (whole-brain family-wise error (FWE) corrected p<0.05, see Table 1 for further statistics).
