# Supplementary figures and images for: Binge drinking differentially affects cortical and subcortical microstructure
Source: Addict Biol. 2017 Jan 20;23(1):403–11. doi: 10.1111/adb.12493 (PMC5811821; doi:10.1111/adb.12493)

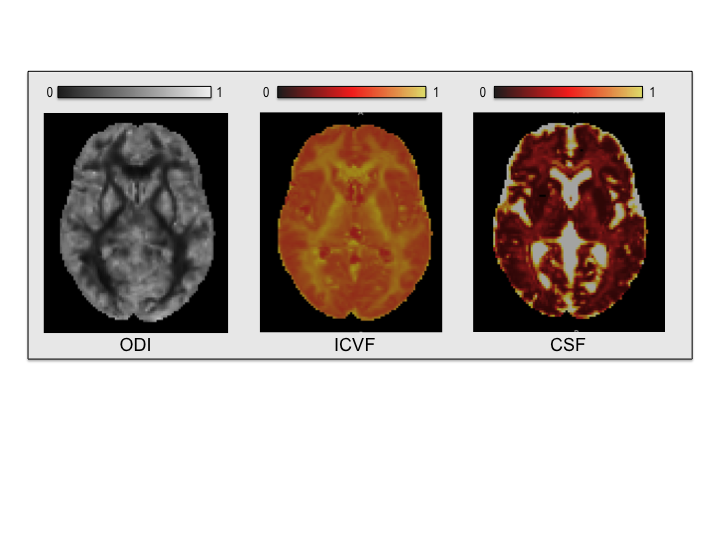

Supplement: Supplementary file 1 — Supporting Info Item [file ADB-23-403-s001.zip › Slide3.tiff]

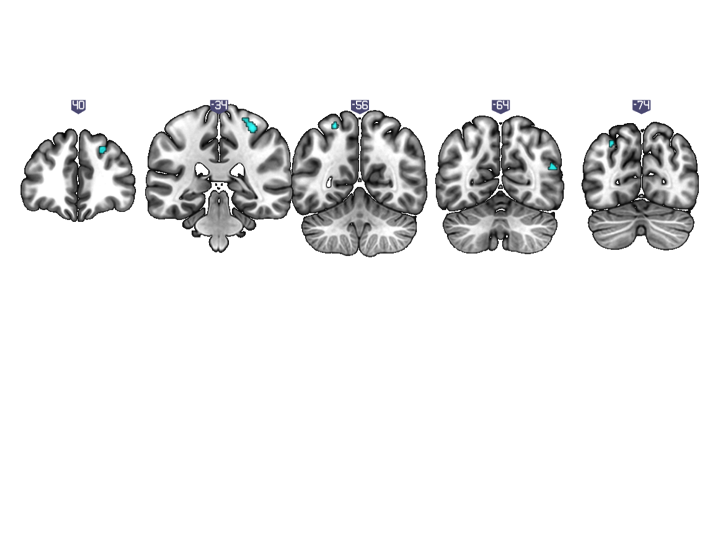

Supplement: Supplementary file 1 — Supporting Info Item [file ADB-23-403-s001.zip › Slide4.tiff]
